# Malignant Transformation of Musculoskeletal Lesions with Imaging–Pathology Correlation—Part 1: Bone Lesions

**DOI:** 10.3390/diagnostics15243120

**Published:** 2025-12-08

**Authors:** Hyang Sook Jeong, Seul Ki Lee, Jee-Young Kim, Changyoung Yoo, Min Wook Joo

**Affiliations:** 1 Department of Hospital Pathology, St. Vincent’s Hospital, College of Medicine, The Catholic University of Korea, Seoul 16247, Republic of Korea; 2 Department of Radiology, St. Vincent’s Hospital, College of Medicine, The Catholic University of Korea, Seoul 16247, Republic of Korea; 3 Department of Orthopaedic Surgery, St. Vincent’s Hospital, College of Medicine, The Catholic University of Korea, Seoul 16247, Republic of Korea

**Keywords:** malignant transformation, bone lesions, imaging findings, pathology, orthopedic oncology

## Abstract

**Background/Objectives:** Malignant transformation of bone lesions, although rare, poses a major diagnostic and clinical challenge. Common benign lesions (e.g., osteochondroma, enchondroma, fibrous dysplasia, giant cell tumor), non-tumorous conditions (e.g., chronic osteomyelitis, irradiated bone, infarction), and low-grade malignancies (e.g., low-grade osteosarcoma, chondrosarcoma) can evolve into aggressive malignancies through distinct genetic, molecular, and microenvironmental pathways. Recognizing early malignant transformation on imaging is crucial for timely diagnosis and management. **Methods:** This review synthesizes current imaging characteristics, pathologic mechanisms, and clinical risk factors associated with malignant transformation of benign and low-grade malignancy bone lesions. **Results:** Atypical imaging findings and inaccurate biopsies can delay diagnosis. Aggressive features—such as cortical destruction, heterogeneous enhancement, and loss of internal matrix—should prompt further pathologic evaluation. Advanced imaging and a multidisciplinary approach with integrated clinicoradiologic–pathologic review are essential to minimize missed diagnoses. Patients with risk factors such as genetic syndromes, prior denosumab therapy, inadequate surgery, or prior radiation therapy require close monitoring or timely intervention. **Conclusions:** Radiologic–pathologic correlation remains central to distinguishing benign from malignant lesions. This review article emphasizes a comprehensive imaging-pathology overview of benign and low-grade malignancy bone lesions with malignant potential, aiming to improve diagnostic accuracy and outcomes in orthopedic oncology.

## 1. Introduction

Common bone lesions, while often benign, can undergo malignant transformation, posing diagnostic challenges. Although typical imaging findings are well established, malignant transformation may present with atypical or subtle imaging findings that are easily overlooked in routine practice. Biopsy may also be limited by sampling error or tumor heterogeneity, underscoring the need for careful imaging–pathology correlation. Early recognition is essential because delayed diagnosis can adversely affect treatment options and patient outcomes. This article highlights the critical importance of malignant transformation of bone lesions and emphasizes that integrating imaging and pathology can enhance diagnostic accuracy and improve outcomes in orthopedic oncology. The goal of this review is to provide practical imaging and pathologic findings, along with surveillance recommendations, for clinicians managing benign and low-grade malignancy bone lesions. This review aims to support radiologists, orthopedic oncologists, and musculoskeletal pathologists in recognizing malignant transformation at earlier and more actionable stages.

### Methodology

This article was designed as a narrative review synthesizing current evidence on the malignant transformation of bone lesions. A literature search included the key terms “malignant transformation”, “secondary sarcoma”, and “dedifferentiation”, relevant to musculoskeletal oncology.

Institutional clinical cases included as illustrative figures were retrospectively identified through the PACS archived from St. Vincent’s Hospital between 2017 and 2024. These cases were selected based on (1) confirmed pathological diagnosis and (2) availability of complete radiologic–pathologic documentation suitable for educational illustration.

All images were anonymized, and only age and sex were retained, as these are considered clinically relevant contextual descriptors. No identifiable patient information was used. Institutional review board approval was obtained solely for the use of de-identified clinical images (Approval No.: VC24RISI0073), and informed consent was waived because no identifiable patient information was used.

This review represents Part 1 of a two-part series and focuses on bone lesions with malignant transformation, while a subsequent part will address soft-tissue lesions.

## 2. Benign Bone Lesions with Malignant Potential: Tumorous Conditions

### 2.1. Osteochondroma

Osteochondroma is a cartilage-capped bony projection continuous with the cortex and medullary cavity of the parent bone, which is best seen on radiographs and computed tomography (CT) [1]. On magnetic resonance imaging (MRI), the cartilage cap appears T2 hyperintense and T1 hypointense, and in adults, it is usually <1–2 cm thick [2].

**Figure 1:**
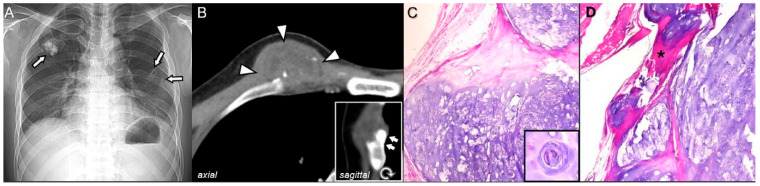
Malignant transformation to secondary peripheral chondrosarcoma (grade 1) of a hereditary multiple exostoses in a 29-year-old man. (**A**) Radiograph of the ribs shows several osteochondromas involving ribs (arrows). (**B**) Axial CT scan of the ribs shows large soft-tissue mass (arrowheads) containing calcifications with cortical and marrow continuity (arrows in inset of sagittal image), representing secondary peripheral chondrosarcoma arising from the cartilage cap. (**C**) Microscopic examination of the rib mass shows a chondroid tumor with irregular lobular architecture, increased cellularity, mild nuclear atypia, and occasional binucleated chondrocyte (inset, original magnification ×400) (H&E, ×20). (**D**) Higher magnification reveals foci of cortical destruction (asterisk) (H&E, original magnification ×200), suggestive of low-grade chondrosarcoma.

Malignant transformation into secondary peripheral chondrosarcoma, although rare, is clinically important. Suspicious clinical signs include progressive pain, post-maturity growth, and soft-tissue swelling. New-onset pain after skeletal maturity is particularly concerning and should prompt further imaging evaluation. Risk is higher in hereditary multiple exostoses (Figure 1, ~10%) than in solitary osteochondromas (~1%) [3]. Malignant transformation often occurs in the proximal femur, proximal humerus, scapula, or pelvis [4]. Molecular studies show that EXT1/EXT2 mutations impair heparan sulfate synthesis, disrupting Indian Hedgehog and BMP signaling and leading to abnormal cartilage proliferation, which contributes to the malignant potential of hereditary multiple exostoses [5].

Imaging features include irregular margins, scattered calcifications within the cartilage cap, and internal lytic areas on radiographs [6]; irregularity in the continuity of cortical and medullary bone on CT (Figure 1) [7]; and cartilage cap thickening > 2 cm in adults (>3 cm in children) with a heterogeneous soft-tissue mass on MRI [2]. Post-gadolinium MRI often demonstrates septal enhancement [4]. Serial imaging follow-up is useful in equivocal cases to detect progressive growth or evolving aggressive features.

Pathological features include disorganized lobulation, active peripheral growth with cortical destruction, soft-tissue invasion, and permeation of the underlying cortex. Additional pathological findings such as increased cellularity, nuclear atypia, binucleated chondrocytes (Figure 1), and myxoid stromal changes may have a subtle appearance in low-grade tumors [8].

### 2.2. Enchondroma

Enchondroma is a benign cartilage-forming tumor that commonly arises in the medullary cavity, typically within the metaphysis or diaphysis [9]. Imaging shows a well-defined, centrally-located lesion with stippled, punctate, or ring-and-arc calcifications. Mild cortical thinning or endosteal scalloping may be present without an aggressive periosteal reaction. CT delineates matrix mineralization and cortical involvement, while MRI demonstrates T2 hyperintensity, T1 hypo- to isointensity, and mild contrast enhancement [4].

Malignant transformation into secondary central chondrosarcoma is uncommon but possible, particularly in Ollier’s disease (10–20%) or Maffucci syndrome (up to 100%) [4]. Worrisome signs include persistent pain, lesion enlargement, or pathologic fracture [4]. Enchondromas commonly harbor IDH1/2 mutations, producing 2-hydroxyglutarate that disrupts DNA methylation and chondrocyte differentiation [10].

**Figure 2:**
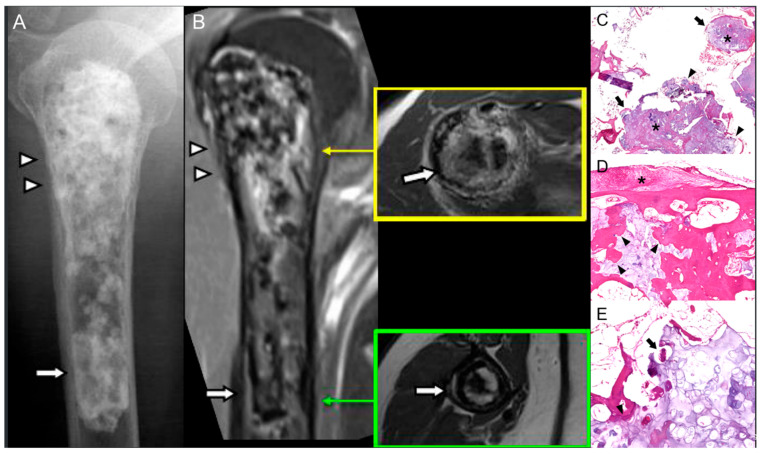
Low-grade chondrosarcomatous transformation of enchondroma in an 81-year-old woman. (**A**) Radiograph of the humerus shows typical radiographic region of an enchondroma (arrow); combined with the features of chondrosarcoma (arrowheads), including (i) extensive endosteal scalloping and (ii) thinning of cortex. (**B**) Coronal MRI scan of enhanced T1 fat-suppressed image shows narrow scalloping (arrow); combined with the features of chondrosarcoma (arrowheads), including (i) extensive endosteal scalloping and (ii) thinning of cortex. Note that axial T2-weighted image at green arrow level shows thin and narrow endosteal scalloping (arrow), and axial image at yellow arrow level shows extensive endosteal scalloping with thin cortex (arrow). (**C**) Microscopic image of the proximal humerus reveals lobulated chondroid nodules (asterisk) in the medullary space, circumscribed by thin rims of bone (arrow) with occasional permeative foci (arrowhead) (H&E, ×100). (**D**) Endosteal scalloping with cartilaginous matrix eroding the cortical bone (arrowhead) and associated cortical thinning and periosteal thickening (asterisk) (H&E, ×10). (**E**) Hyaline cartilage matrix shows increased chondrocyte cellularity, permeates (arrowhead), and entraps (arrow) host bone trabeculae (H&E, ×400), findings compatible with low-grade chondrosarcoma.

Radiologic indicators of malignant transformation include cortical thickening, extensive endosteal scalloping, and cortical destruction (Figure 2) [4]. A lesion larger than 5 cm in the long bones may indicate malignancy. Additionally, changes in the calcification pattern, such as irregular, cloud-like calcifications, are also suspicious for malignant transformation [11]. Heterogeneous signal intensity (SI) on MRI, along with nodular or irregular contrast enhancement, is also indicative of malignant transformation [4]. Comparison with prior imaging is essential because subtle progression over time is often the earliest radiologic clue.

Pathologically, malignant transformation is marked by tumor infiltration into trabecular and cortical bone, invasion of Haversian systems, marrow fat replacement, and fibrous desmoplastic response (Figure 2). Tumor cells demonstrate anaplasia with pleomorphism, hyperchromasia, increased mitoses, and loss of normal cartilage architecture [11].

### 2.3. Giant Cell Tumor of Bone

Giant cell tumor of bone (GCTB) is a benign but locally aggressive bone tumor that predominantly arises in the meta-epiphysis of long bones [12]. Imaging shows a well-defined, expansile, eccentric osteolytic lesion without sclerotic margins or matrix mineralization. MRI demonstrates T2 hyperintensity often with cystic or hemorrhagic areas and low-to-iso T1 signal reflecting cellular and fibrous components [12].

**Figure 3:**
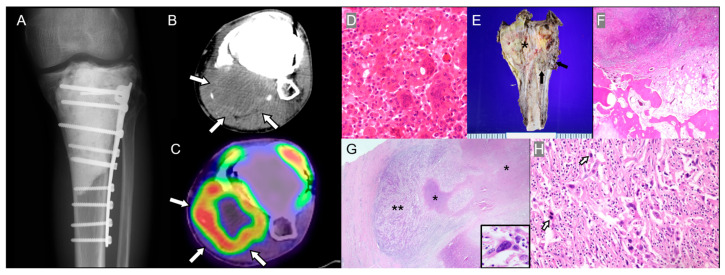
Secondary malignant giant cell tumor of bone (GCTB) in a 40-year-old man. (**A**) History of curettage and cementation with fixation of the left proximal tibia was performed 10 years ago, diagnosed as GCTB. (**B**) Axial CT scan with enhancement shows a large extraosseous mass at the site of previous surgery (arrows). (**C**) ^18^F-FDG PET/CT shows increased uptake in the popliteal fossa (arrows). (**D**) Prior biopsy of the proximal tibial lesion demonstrates giant cell tumor of bone (H&E, ×400). (**E**) Gross specimen after limb-salvage surgery for the recurrent tibial lesion shows an infiltrative mass involving the proximal tibia (asterisk) with an extraosseous soft-tissue component (arrow). (**F**) Low-power histologic section reveals replacement of the medullary space by tumor cells with permeative growth (H&E, ×40). (**G**) Extraosseous soft-tissue mass with infiltrative tumor cells (double asterisks) containing atypical spindle cells with marked nuclear atypia (inset, original magnification, ×650) and geographic necrosis (asterisk) (H&E, ×10). (**H**) Higher magnification reveals features of a high-grade sarcoma with pleomorphic spindle cells and elevated mitotic activity (arrows; 12/10 HPFs), including atypical forms (H&E, ×400), consistent with undifferentiated pleomorphic sarcoma.

Malignant transformation is rare and may occur primarily or secondarily after treatment such as radiation or surgery [13]. Primary malignant GCTB contains both benign and sarcomatous components at diagnosis, whereas secondary malignant GCTB develops at a previously treated site after a long latency, often up to 10 years (Figure 3) [13]. Recent genomic profiling suggests that secondary malignant GCTBs may acquire additional TP53 or TERT promoter mutations, indicating clonal evolution toward high-grade sarcoma [14]. New or rapidly increasing pain after initial treatment should raise suspicion for secondary malignant transformation, even if imaging findings are subtle.

The Campanacci grading system classifies GCTB by radiological aggressiveness as Grade 1 (latent), well-defined margins, intact cortex; Grade 2 (active), cortical thinning and slight expansion; or Grade 3 (aggressive), cortical destruction, soft-tissue extension [15]. Malignant transformation shows more aggressive findings, such as cortical destruction, periosteal reactions, and soft-tissue masses (Figure 3) [13].

Pathologically, malignant transformation features high-grade sarcomatous components (osteosarcoma, fibrosarcoma, or undifferentiated pleomorphic sarcoma [UPS]) [16]. In primary malignant GCTB, benign and malignant elements coexist, while secondary malignant GCTB may entirely lose conventional GCTB features to resemble other primary high-grade bone sarcomas (Figure 3) [16].

### 2.4. Fibrous Dysplasia

Fibrous dysplasia is a benign lesion in which normal bone is replaced by fibrous tissue and immature bone, typically showing a ground-glass appearance [17]. It may be monostotic or polyostotic and appears as a well-defined, sometimes expansile medullary lesion with cortical thinning but no cortical breakage. On MRI, it demonstrates intermediate-to-low T1 and intermediate-to-high T2 SI [17].

Malignant transformation is rare but may result in fibrosarcoma, osteosarcoma, or UPS. GNAS mutations cause constitutive Gsα–cAMP activation, driving persistent osteoblastic signaling and a predisposition to malignant osteoid formation, especially after radiation exposure [18,19]. Clinical warning signs include new or worsening pain, rapid swelling, or a palpable mass at the lesion site [17].

Radiologically, malignant transformation is suggested by the loss of ground-glass appearance, development of heterogeneous lytic or sclerotic areas, cortical destruction, and periosteal reaction. On MRI, malignant lesions often exhibit high T1 SI (cellularity or hemorrhage) and heterogeneous T2 SI with necrosis [17,20]. The appearance of a soft-tissue mass or cortical breakthrough on follow-up imaging is particularly concerning and warrants biopsy.

Pathologically, malignant transformation features marked cellularity, pleomorphism, increased mitosis, and loss of normal fibrous and immature bone architecture with abnormal osteoid or chondroid matrix production [18].

### 2.5. Liposclerosing Myxofibrous Tumor of Bone

Liposclerosing myxofibrous tumor (LSMFT) is a rare fibro-osseous lesion that predominantly occurs in the intertrochanteric region of the femur (80–90%) and is often discovered incidentally [21]. Radiographs and CT typically show a well-defined geographic lytic lesion with sclerotic margins, sometimes containing a mineralized matrix [22]. MRI reveals heterogeneous SI: low-to-intermediate on T1WI (fibrous tissue) and high SI on T2WI (myxoid component). Small amounts of fat may be present but are usually indistinct on T1WI [21].

Malignant transformation is uncommon but has been reported (up to 10–16% in small retrospective series and case reports) [21,23,24]. The debate persists on whether LSMFT is a distinct disease entity or a secondary change in other benign lesions, such as fibrous dysplasia or intraosseous lipoma, which could explain variable incidence rates [21]. At the molecular level, recent studies have identified occasional GNAS and MDM2/CDK4 amplifications in LSMFT, suggesting overlap with fibrous dysplasia and low-grade central osteosarcoma (LGCOS) [25]. Nonetheless, rapid recurrence or disproportionate pain after surgery should raise suspicion of malignancy [23].

Radiologic findings suggestive of malignant transformation include rapid lesion growth, cortical destruction with soft-tissue extension, aggressive periosteal reaction, and new intense enhancement on contrast-enhanced MRI [23,26].

Pathologically, malignant transformation is often associated with ischemic ossification and usually results in osteosarcoma, and less commonly UPS [23].

## 3. Benign Bone Lesions with Malignant Potential: Non-Tumorous Conditions

### 3.1. Chronic Osteomyelitis

Chronic osteomyelitis is a persistent bone infection that often follows inadequately treated acute osteomyelitis. Radiographs typically reveal sequestrum (necrotic bone), involucrum (new bone formation), and cloaca (drainage sinus), along with sclerosis and cortical thickening [27,28]. CT provides detailed visualization of sequestra, involucra, cortical destruction, and may show intraosseous gas [27,28]. MRI is sensitive for detecting marrow and soft-tissue changes, revealing low T1, high T2 SI, and intense enhancement [27,28].

**Figure 4:**
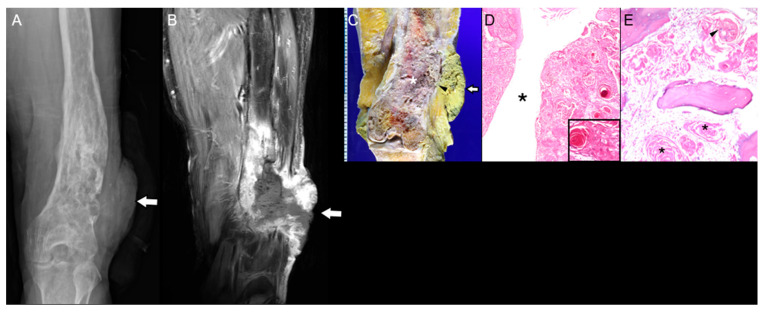
Marjolin’s ulcer in a 66-year-old man with a history of surgical management of chronic osteomyelitis 36 years ago. (**A**) Plain radiograph shows soft-tissue mass (arrow) at the lateral side of thigh. Extensive bone destruction is associated. (**B**) Coronal T1-weighted enhanced MRI shows an extensive ulcerating mass (arrow) spreading to adjacent areas of the meta-diaphysis (asterisk) with enhancement. (**C**) Gross cut surface of the left thigh shows an exophytic cutaneous mass (arrow) involving the dermis and subcutis, continuous via a cortex-breaking sinus tract (arrowhead) with a gray–white cavitary lesion (asterisk) in the medullary space of the underlying bone. (**D**) Mid-power photomicrograph demonstrates squamous cell carcinoma arising along the skin sinus tract (asterisk), with invasive nests and keratinization (inset, ×400), in a background of chronic inflammation (H&E, ×200). (**E**) Medullary space is replaced by squamous carcinoma cells (arrowhead) with keratin pearls (asterisk) (H&E, ×400).

A serious late complication is Marjolin’s ulcer, a squamous cell carcinoma (SCC) arising in a chronic sinus tract, typically after a latency of 20–30 years (Figure 4) [29,30]. Clinically, malignant transformation presents with new pain, increased or bloody discharge, foul odor, and regional lymphadenopathy [31]. Persistent sinus formation despite appropriate antibiotic therapy should raise clinical suspicion for malignant degeneration. At the molecular level, chronic inflammation drives persistent ROS and cytokine release (TNF-α, IL-6), causing DNA damage and epithelial proliferation. Marjolin’s ulcer often shows TP53 mutation, CDKN2A loss, and EGFR overexpression, consistent with inflammation-induced carcinogenesis [32,33].

Radiologic features of malignant transformation include irregular osteolysis, mixed sclerotic–lytic changes, aggressive periosteal reaction, and, on CT/MRI, a heterogeneously enhancing soft-tissue mass with cortical destruction, marrow invasion, and loss of fat planes (Figure 4). Progressive bone destruction around a chronic sinus tract on serial imaging strongly suggests malignant transformation rather than recurrent infection.

Pathologically, Marjolin’s ulcer with invasive SCC typically arises from the epithelium lining a chronic sinus tract or the base of a long-standing ulcer. It is characterized by atypical keratinocytes, keratin pearls, and infiltrative growth into adjacent tissues, often accompanied by chronic inflammation and fibrosis, reflecting long-standing infection (Figure 4) [34].

### 3.2. Bone That Underwent Radiation Therapy

Radiation therapy can cause characteristic structural and vascular changes in bone, including diffuse osteopenia or osteoporosis, patchy sclerosis, trabecular disorganization, and cortical thinning, sometimes leading to pathologic fractures in weight-bearing regions [35]. On MRI, irradiated bone shows low T1 SI due to fatty replacement or fibrosis, variable T2 signal depending on edema or sclerosis, and minimal enhancement due to poor vascularity [35]. These post-treatment changes may mimic tumor recurrence, making careful comparison with prior imaging essential.

**Figure 5:**
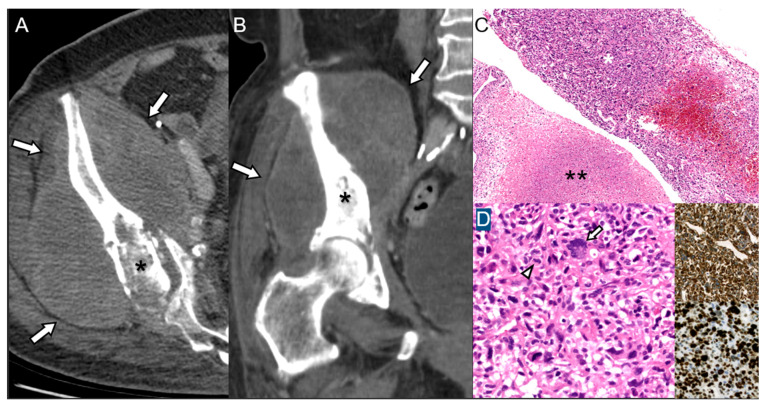
Radiation-induced osteosarcoma of ilium in a 73-year-old woman 16 years after radiation therapy for endometrial cancer. (**A**) Axial and (**B**) coronal CT scans show a bony destruction of right ilium (asterisks) with large soft-tissue mass (arrows). (**C**) Needle biopsy of the pelvic tumor demonstrates a highly cellular spindle cell proliferation (asterisk) with geographic tumor necrosis (double asterisks) (H&E, ×20). (**D**) High-power view shows pleomorphic spindle cells, occasional tumor giant cells (arrow), increased mitotic activity (8/10 HPFs; arrowhead), diffuse vimentin expression (inset, top), and an elevated Ki-67 proliferation index (95%; inset, bottom), consistent with high-grade sarcoma, most consistent with conventional osteosarcoma (H&E, ×200; insets, original magnification ×40).

Radiation-induced sarcoma (RIS) is a rare but serious late complication, typically arising 2 to 21 years after exposure to doses >3000 cGy (Figure 5) [36]. According to Cahan criteria [37], RIS must develop within the irradiated field, show sarcomatous histology, and be absent prior to radiation. Clinically, patients present with new or worsening pain, rapidly enlarging masses, or pathological fractures in the irradiated area [38]. At the molecular level, radiation-induced sarcomas commonly show TP53 and RB1 mutations with complex chromosomal rearrangements, reflecting radiation-driven genomic instability [39,40,41].

Radiologically, RIS appears as a new, aggressive lytic lesion within previously sclerotic bone, often with soft-tissue extension, cortical destruction, and aggressive periosteal reaction (Figure 5) [42]. MRI demonstrates heterogeneous enhancement, cortical breakthrough, and expansile growth (Figure 5) [42]. The development of a new soft-tissue mass in the radiation field is a key imaging feature that should prompt biopsy to exclude RIS.

Pathologically, RIS most commonly manifests as high-grade osteosarcoma (Figure 5) or UPS, though chondrosarcoma and angiosarcoma can occur [43]. Tumors exhibit marked cellular atypia, pleomorphism, high mitotic activity, necrosis, and matrix production (osteoid, cartilage, or collagen), consistent with highly malignant behavior (Figure 5) [43].

### 3.3. Bone Infarction

Bone infarction, or medullary/metaphyseal infarct, results from ischemic necrosis of the bone marrow due to factors such as trauma, corticosteroid use, sickle cell disease, or embolic events [44]. It is often asymptomatic and incidentally discovered. Radiographs show a serpiginous sclerotic border outlining a central lucency, while MRI demonstrates the characteristic double-line sign on T2WI with smooth, well-defined margins [44].

**Figure 6:**
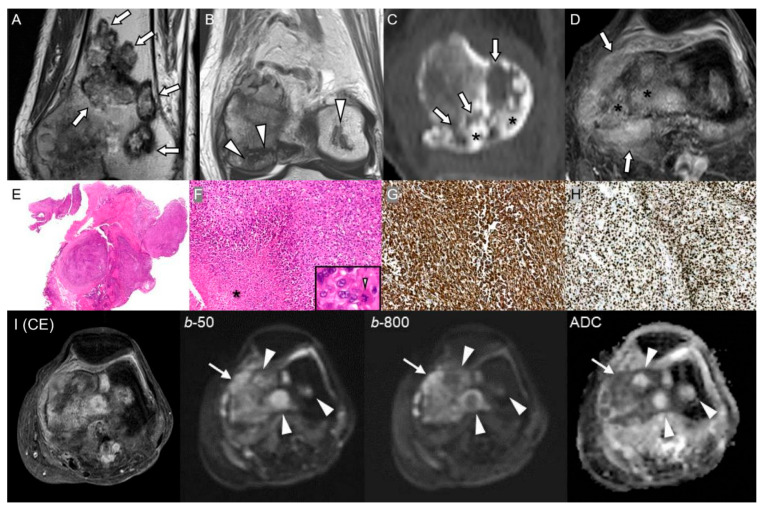
Malignant transformation of a distal femoral area of osteonecrosis in an 81-year-old woman. (**A**,**B**) Coronal T2-weighted MR images show multiple areas of bone infarction (arrows) and osteonecrosis (arrowheads) in the distal femoral metadiaphysis. (**C**) Sagittal CT scan shows a radiodense area of osteonecrosis (asterisks) and surrounding aggressive bone destruction with osteolysis (arrows). (**D**) Axial T1-weighted fat-suppressed enhanced MR image shows marrow replacing lesions (asterisks) with extraosseous soft-tissue mass formation (arrows). (**E**) Extraosseous component of the distal femur mass invading subcutis and surrounding soft tissue (H&E, ×5). (**F**) High-power view demonstrates highly cellular, pleomorphic tumor cells with nuclear atypia, increased mitoses (9/10 HPFs), including atypical forms (inset, arrowhead, original magnification ×650), and tumor necrosis (asterisk) (H&E, ×150). (**G**,**H**) Diffuse vimentin expression and markedly increased Ki-67 labeling index 95% (×200, respectively), compatible with high-grade sarcoma, conventional type osteosarcoma. (**I**) On axial T1-weighted fat-suppressed enhanced MRI, both osteonecrosis and malignant transformation show enhancement, making differentiation challenging. On diffusion-weighted imaging, osteonecrosis areas (arrowheads on *b*-50, *b*-800, and ADC map) do not exhibit diffusion restriction, while regions of malignant transformation (arrows on *b*-50, *b*-800, and ADC map) demonstrate diffusion restriction, guiding precise biopsy for definitive diagnosis.

Malignant transformation is rare, and the true incidence remains uncertain, but it is suspected when new or progressive pain, swelling, or a palpable mass develops at a previously stable infarct, typically after a 10–20-year latency [45]. Transformation risk increases in metadiaphyseal or multifocal infarctions, most commonly around the knee (Figure 6) [45]. At the molecular level, chronic ischemia-induced oxidative stress causes DNA damage and TP53 pathway disruption. Occasional MDM2 or CDK4 amplification suggests a shared mechanism with other secondary osteogenic sarcomas [46].

Radiologic clues include loss of the smooth sclerotic rim, new lytic or destructive lesions, focal osteolysis, cortical breakthrough, periosteal reaction, and soft-tissue extension [47]. MRI reveals heterogeneous signals, post-contrast enhancement, and mass-like marrow replacement around areas of osteonecrosis with poorly defined margins (Figure 6) [48,49].

Pathologically, transformation usually produces high-grade sarcomas, most frequently UPS, followed by osteosarcoma, fibrosarcoma, and angiosarcoma [50]. These arise at the reactive interface of the infarct, showing atypical spindle cells, high mitotic activity, osteoid or fibrous matrix production, and infiltrative growth with necrosis and hemorrhage, reflecting aggressive malignancy (Figure 6) [51].

### 3.4. Paget’s Disease of Bone

Paget’s disease of bone is a chronic disorder characterized by excessive and disorganized bone remodeling, showing a spectrum from lysis to sclerosis [52]. In its advanced stage, imaging reveals cortical thickening, bone expansion, and coarsened trabeculae, leading to bone enlargement and deformity [53]. The classic appearance includes the “cotton wool” skull, “ivory vertebra”, and “brim sign” of the pelvis [54]. Radiographs best demonstrate structural changes, while bone scans assess the extent and activity [54].

**Figure 7:**
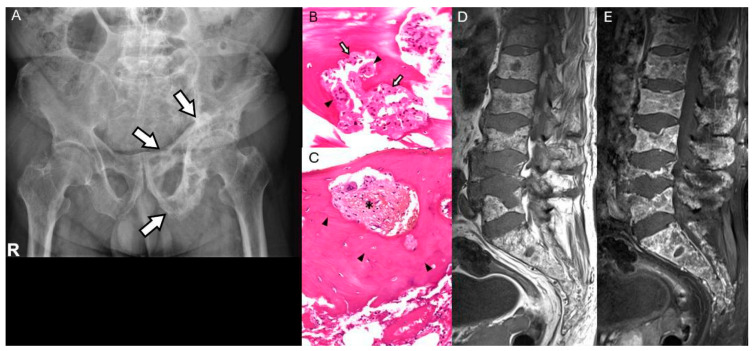
Paget’s disease of pelvis in a 73-year-old man. (**A**) Radiograph of the pelvis shows cortical thickening of the left iliopectineal line and ilioischial lines (arrows) with trabecular thickening, as compared to the right side. (**B**) High-power photomicrograph reveals woven bone trabeculae showing both osteoblastic (arrow) and osteoclastic (arrowhead) activity, forming areas of bone resorption (“bite marks”) (H&E, ×400). (**C**) Osteolytic lacunae with irregular cement lines (arrowhead) demarcating lamellar bone, accompanied by increased peritrabecular fibrosis (asterisk), consistent with Paget’s disease (H&E, ×200). (**D**,**E**) Sagittal T1 non-contrast and contrast MR images show a salt-and-pepper pattern in the bone marrow, indicative of possible multiple myeloma involvement, confirmed as CRAB(+/−/+/+), ISS I, IgG, κ type.

Sarcomatous transformation is a rare but severe complication, occurring in about 1% of cases (this percentage is based on historical institutional series), typically in polyostotic disease and late stages [55,56]. Clinically, it presents with new focal bone pain, swelling, or mass formation [57]. At the molecular level, mutations in SQSTM1, TNFRSF11A, or ZNF687 activate NF-κB signaling and disrupt osteoclast regulation. Additional TP53 mutations or MDM2 amplification may drive sarcomatous transformation in advanced disease [58,59]. Occasionally, giant cell tumors or hematologic malignancies (e.g., multiple myeloma, leukemia, and lymphoma) can coexist with or mimic sarcomatous changes (Figure 7) [60].

Radiologically, osteosarcoma secondary to Paget’s disease appears as an aggressive lytic lesion with cortical destruction and a soft-tissue mass [53]. Giant cell tumors are lytic but lack aggressive periosteal reaction, while hematologic malignancies may show multiple lytic or sclerotic lesions, sometimes in non-pagetic bone, helping distinguish them from sarcoma (Figure 7) [53,61]. Rapid change from long-standing stable disease or asymmetric cortical destruction is a key imaging clue for malignant transformation.

Pathologically, osteosarcoma shows pleomorphic malignant cells, high mitotic activity, and malignant osteoid production with cortical invasion and soft-tissue extension [62]. In contrast, giant cell tumors feature osteoclast-like giant cells with mononuclear stromal cells and minimal atypia, and hematologic malignancies show marrow infiltration by atypical plasma, blast, or lymphoid cells, producing variable bone destruction patterns, affecting both pagetic and non-pagetic bones (Figure 7) [62].

## 4. Low-Grade Malignancies into Higher-Grades

### 4.1. Low-Grade Central Osteosarcoma

**Figure 8:**
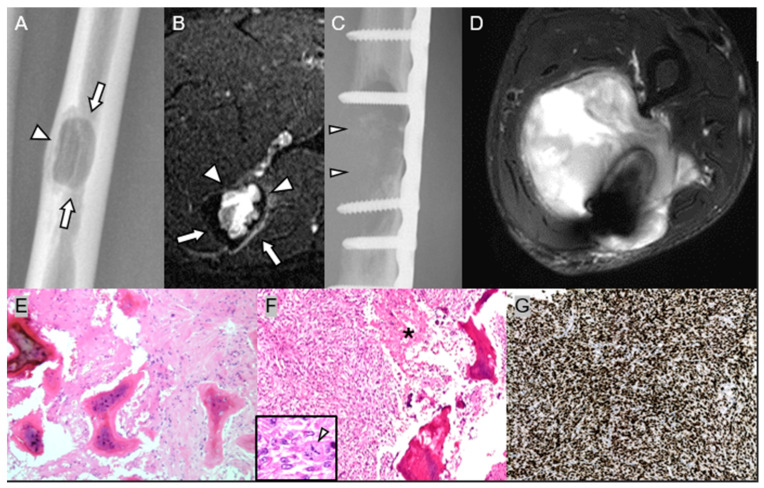
High-grade dedifferentiation of a low-grade central osteosarcoma of ulnar shaft in a 52-year-old man. (**A**,**B**) Initial plain radiograph and axial T2-weighted fat-suppressed MR image show well-defined osteolytic lesion in the medullary part of ulnar shaft (arrows) with cortical involvement (arrowheads), initially diagnosed as fibrous dysplasia; however, a retrospective pathology review suggested the possibility of low-grade central osteosarcoma. (**C**,**D**) Post-operative plain radiograph and axial T2-weighted fat-suppressed MR image after extended curettage with internal fixation show cortical destruction (arrowheads) with extraosseous soft-tissue mass formation. (**E**) Initial curettage specimen shows branching trabeculae of woven bone within a bland fibrous stroma, diagnosed as fibrous dysplasia (H&E, ×200). (**F**) Wide excision specimen of the recurrent ulnar mass obtained 7 months later demonstrates highly cellular, pleomorphic spindle cells with tumor necrosis (asterisk), and frequent mitoses (46/10 HPFs), including atypical forms (inset, arrowhead; original magnification ×650) (H&E, ×200). (**G**) Ki-67 proliferation index up to 99% (×200), consistent with high-grade sarcoma, diagnosed as fibroblastic osteosarcoma. A retrospective pathological review of the unusual course of this case suggests the possibility of underdiagnosis in the initial biopsy, with potential dedifferentiation from low-grade to high-grade osteosarcoma. This may have resulted from undersampling of low-grade osteosarcoma tissue during the initial curettage. Microscopically, distinguishing between fibrous dysplasia and low-grade osteosarcoma is challenging in the absence of clear evidence of bone permeation.

LGCOS is a rare, slow-growing intramedullary tumor usually arising in the metaphyseal or metadiaphyseal region of long bones (femur or tibia). Radiographically, it often mimics fibrous dysplasia, showing a well-defined mixed lytic–sclerotic lesion with bone expansion, cortical thinning, and sometimes chronic periosteal thickening (Figure 8) [63]. Unlike fibrous dysplasia, LGCOS may exhibit variable osteoid matrix mineralization, which is best visualized on CT [64]. On MRI, it demonstrates low-to-intermediate T1 and heterogeneous high T2 SI, confirming its intramedullary origin and extent [64].

Dedifferentiation into a high-grade osteosarcoma can occur, especially after incomplete resection or curettage (Figure 8) [65]. Clinical warning signs include new or progressive pain, swelling, or a palpable mass at the prior tumor site [65]. At the molecular level, LGCOS and related subtypes show MDM2 and CDK4 gene amplification on chromosome 12q13–15, leading to p53 and RB1 pathway inactivation. Dedifferentiation is linked to additional TP53 mutations and genomic instability, driving increased proliferation and aggressive transformation [66,67,68,69].

Radiologically, high-grade progression (dedifferentiation) manifests as progression from sclerotic to lytic, permeative destruction, cortical breakthrough, new periosteal reactions, and soft-tissue extension, with loss of clear margins indicating aggressive invasion (Figure 8) [65]. New aggressive radiologic features in a previously stable lesion should prompt biopsy to exclude dedifferentiation.

Histologically, dedifferentiation is characterized by a transition from bland spindle cells to highly pleomorphic, mitotically active malignant cells producing disorganized osteoid (Figure 8). Loss of MDM2 and CDK4 expression may accompany this shift, signifying molecular progression [70]. Low-grade osteosarcomas—including central, parosteal, and periosteal subtypes—share this potential for high-grade dedifferentiation, particularly following recurrence or incomplete excision [71].

### 4.2. Conventional Low-Grade Chondrosarcoma

Conventional low-grade chondrosarcoma (Grade I) arises within the medullary cavity of the long bones (femur or humerus) or flat bones (pelvis or ribs), either de novo or from preexisting lesions such as enchondroma or osteochondroma. Radiographs and CT show a central, lobulated intramedullary lesion with ring-and-arc or popcorn-like calcifications, endosteal scalloping (often >2/3 of cortical thickness), and cortical thinning or mild expansion without aggressive periosteal reaction [4]. MRI demonstrates low-to-intermediate T1 and high T2 SI with septal or peripheral enhancement, reflecting its cartilaginous and low-grade nature [72].

Dedifferentiation into a high-grade sarcoma (most often UPS or osteosarcoma) is rare but serious, particularly in central lesions of the long bones and pelvis [73]. Clinically, it presents with sudden worsening pain, swelling, or rapid lesion enlargement, and sometimes, pathologic fracture or systemic symptoms [73]. The appearance of metastasis, especially to the lungs, confirms high-grade progression (dedifferentiation) [74]. At the molecular level, conventional chondrosarcomas commonly harbor IDH1 or IDH2 mutations that generate 2-hydroxyglutarate, leading to epigenetic dysregulation and impaired chondrocyte differentiation. Dedifferentiated forms often acquire TP53 or CDKN2A alterations and genomic instability, driving progression to a high-grade phenotype [75,76,77,78].

**Figure 9:**
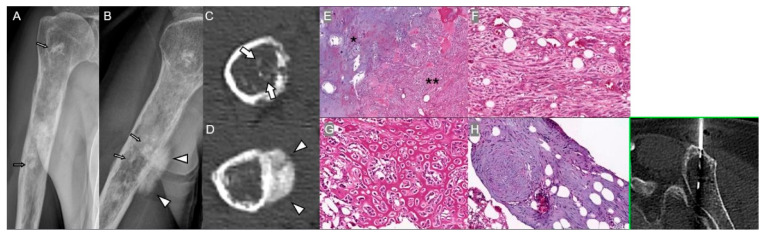
Dedifferentiated chondrosarcoma with multiple bone metastasis in spine and pelvic bone in a 54-year-old man. (**A**,**B**) Plain radiographs of right humerus show an extensive, poorly defined mixed lytic and sclerotic lesion in the proximal humerus with endosteal scalloping. Note the cartilaginous mineralization in the intramedullary cavity (arrows) and the densely osteoblastic mineralization of the surface (arrowheads). (**C**,**D**) Axial CT scans clearly depict the chondral-type mineralization (arrows) and the densely osteoblastic soft-tissue mass (arrowhead). (**E**) Humeral intramedullary mass composed of low-grade central chondrosarcoma (asterisk) and high-grade non-cartilaginous sarcoma (double asterisks) with abrupt juxtaposition (H&E, ×70). The high-grade component demonstrates (**F**) pleomorphic spindle cell proliferation and (**G**) delicate osteoid formation, consistent with conventional osteosarcoma (H&E, ×200, respectively). (**H**) Pelvic metastatic lesion shows morphology comparable to the spindle cell component of the humeral mass (H&E, ×100). Next-generation sequencing demonstrates an IDH2 mutation, supporting a diagnosis of metastatic dedifferentiated chondrosarcoma.

Radiologically, dedifferentiated chondrosarcoma shows tumor bimorphism—coexisting features of low- and high-grade components within the same lesion [74]. Findings include loss of defined borders, cortical destruction, soft-tissue extension, aggressive periosteal reaction, and loss of chondroid matrix (Figure 9) [74]. MRI reveals solid, enhancing tissue replacing lobulated high T2 SI, consistent with dedifferentiation [74]. Rapid development of a soft-tissue mass or cortical breach in a previously stable lesion should raise concerns about high-grade progression (dedifferentiation).

Pathologically, it exhibits biphasic morphology: a low-grade cartilaginous component with mild atypia adjacent to a high-grade non-cartilaginous sarcomatous component (commonly UPS) [72]. The high-grade component shows marked pleomorphism, atypical mitosis, necrosis, and loss of chondroid matrix (Figure 9). Both low-grade central and peripheral chondrosarcomas can undergo this high-grade dedifferentiation [79].

Table 1 summarizes the characteristic clinical, imaging, and histopathologic findings of malignant transformation.

## 5. Precise Biopsy for Accurate Pathologic Diagnosis

Image-guided biopsy plays a key role in confirming malignant transformation. Combining functional imaging techniques—such as diffusion-weighted imaging (DWI), dynamic enhanced MRI, and even positron emission tomography—can improve target selection by distinguishing aggressive malignant lesions (e.g., diffusion restriction) from benign processes (such as necrosis or fibrosis, Figure 6) [80]. Biopsy planning is essential and should be performed in consultation with the orthopedic oncologic surgeon to ensure that the biopsy tract is positioned along a potential surgical approach to avoid tumor contamination of unaffected compartments. The biopsy path should avoid neurovascular bundles and joints, and traverse a single anatomic compartment whenever possible [81].

A multidisciplinary approach involving radiologists, pathologists, and orthopedic oncologists is vital to plan and perform image-guided biopsies. Obtaining multiple, representative tissue samples is critical, especially when the initial pathological diagnosis (e.g., low-to-intermediate grade chondrosarcoma) does not align with aggressive clinical behavior such as unexpected metastasis (Figure 9). Repeat biopsy should be considered when histologic findings are inconsistent with imaging or clinical suspicion, as sampling error and tumor heterogeneity may lead to false-negative results. In such cases, thorough clinicoradiologic–pathologic correlation may reveal a revised diagnosis, for instance, dedifferentiated chondrosarcoma, which profoundly impacts prognosis and treatment strategy.

## 6. Patients at Risk for Malignant Transformation

Recognizing patients at high risk for malignant transformation is crucial for timely intervention. Key risk groups include the following: (i) Patients with genetic syndromes such as multiple hereditary exostoses, Ollier disease, and Maffucci syndrome [4]; (ii) patients previously treated with denosumab for GCTB, as reports indicate potential transformation into high-grade sarcomas, such as osteosarcoma, requiring close surveillance [82]; (iii) patients with prior inadequate surgery, such as marginal excision of low-grade central osteosarcoma, who are prone to local recurrence and dedifferentiation, often requiring wide excision for tumor control [83]; and (iv) patients with a history of radiation therapy, a well-established cause of RIS arising years after radiotherapy for unrelated malignancies [84].

Table 2 summarizes genetic syndromes-, treatment-, surgery-, and radiation-related factors predisposing to malignant transformation. Awareness of these conditions allows early detection through targeted surveillance and timely biopsy when new symptoms arise. Surveillance should be tailored to individual risk profiles, with high-risk patients undergoing periodic clinical evaluation and interval imaging. For lesions with established malignant potential, MRI is preferred for local assessment, while CT or radiography may be used in long-term monitoring of stable lesions. PET-CT may be added when malignant transformation is suspected or when symptoms progress despite inconclusive conventional imaging. Regular follow-up, typically every 6–12 months, depending on risk category and symptom evolution, enables early detection of radiologic changes and supports timely intervention [85,86].

Surveillance strategies should combine periodic imaging and clinical assessment, but management must also consider appropriate surgical timing. Lesions that show radiologic progression, new soft-tissue extension, or biopsy-proven dedifferentiation require prompt referral to orthopedic oncology for surgical planning. When malignancy is confirmed or strongly suspected, wide excision with negative margins is preferred over intralesional curettage to reduce the risk of recurrence and metastatic spread [87]. In anatomically complex regions, limb-sparing surgery is achievable in many cases with careful preoperative planning, while amputation is reserved only for unresectable or functionally devastating disease. Surgical decisions should always integrate oncologic principles, including en bloc resection and biopsy tract excision when applicable [81]. Early multidisciplinary consultation is essential to avoid delays in definitive surgery and to optimize functional outcomes.

**Figure 10:**
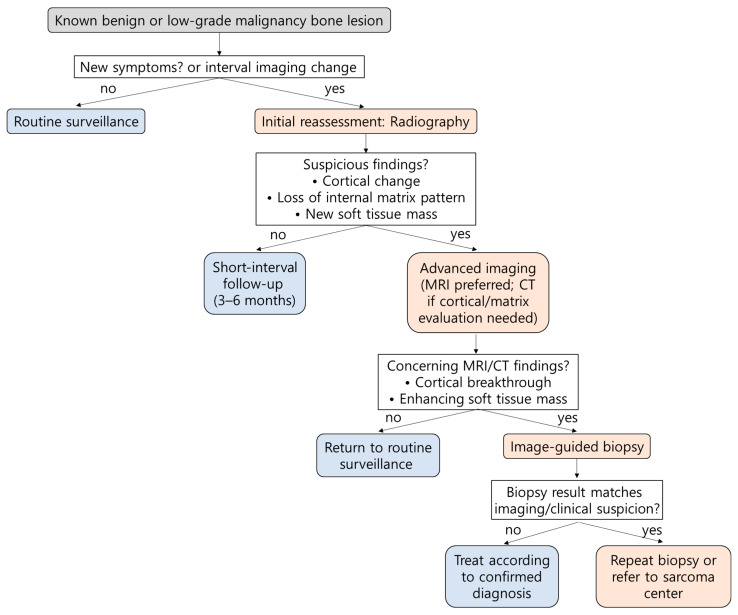
Clinical workflow for suspected malignant transformation in benign or low-grade malignancy bone lesions. This flowchart summarizes the recommended stepwise approach to evaluating suspected malignant transformation in previously diagnosed benign or low-grade malignancy bone lesions.

Figure 10 provides an algorithm of a stepwise approach to evaluating suspected malignant transformation in previously benign or low-grade malignancy bone lesions, guiding when to escalate imaging, perform biopsy, or initiate referral.

## 7. Conclusions

Malignant transformation of bone lesions is a rare but serious event that exists as a biological continuum, linking genetic predisposition, microenvironmental stress, and therapeutic exposure. Its diagnosis can be challenging due to the absence of distinct diagnostic indicators. Despite its rarity, early recognition is crucial, as delayed diagnosis can negatively affect patient prognosis and treatment planning. Integrating imaging, pathology, and clinical history is essential for accurate assessment. Radiologists play a central role not only in identifying the imaging hallmarks of transformation but also in guiding biopsy and follow-up strategies. A structured, multidisciplinary approach combining radiologic insight, pathologic validation, and clinical context can prevent delayed diagnosis and optimize outcomes.

Close surveillance is particularly important in patients with known predisposing factors such as hereditary exostosis syndromes, prior radiation therapy, inadequate previous surgery, or denosumab exposure. Recognition of interval change remains a key principle in musculoskeletal oncology, and comparison with prior imaging should be routine practice, especially when evaluating lesions with indeterminate features.

Future directions should focus on improving diagnostic precision through the incorporation of quantitative imaging biomarkers, radiomics-based analysis, and radiogenomic correlation. These emerging techniques have the potential to detect early malignant transformation by characterizing intratumoral heterogeneity beyond conventional imaging. Additionally, standardized surveillance protocols and risk-based management guidelines are needed to support consistent clinical decision-making across institutions. Improved collaboration among radiology, orthopedic oncology, pathology, and molecular genetics will continue to advance individualized patient care and contribute to better long-term outcomes.

## Figures and Tables

**Table 1:** Key Clinical, Imaging, and Pathologic Indicators of Malignant Transformation.

Lesion	Key Clinical Indicators	Imaging Red Flags	Pathologic Correlates
Osteochondroma	New or progressive pain, growth after skeletal maturity; usually long-standing lesion (>10 yr)	Cartilage cap > 2 cm (adult), irregular or lobulated contour, soft-tissue mass	Increased cellularity, binucleated chondrocytes, cortical invasion
Enchondroma	Persistent pain, enlargement, fracture; risk ↑ in Ollier’s (10–20%) and Maffucci (up to 100%)	Endosteal scalloping > 2/3 cortex, cortical thickening or destruction, irregular calcifications	Trabecular permeation increased atypia, marrow fat replacement
Giant Cell Tumor of Bone	May occur primarily or 10 yr post-surgery/radiation; Recurrence, rapid symptoms post-treatment	Cortical destruction, new soft-tissue extension, aggressive periosteal reaction	Sarcomatous spindle transformation, high mitotic activity
Fibrous Dysplasia	Rapid swelling, new pain, post-radiation change	Loss of ground-glass pattern, mixed sclerotic/lytic change, cortical breach	High-grade atypia, malignant osteoid/chondroid matrix
Liposclerosing Myxofibrous Tumor	Transformation latency 5–15 yr; new pain or recurrence after curettage	Rapid lesion growth, soft-tissue extension, heterogeneous enhancement	Osteoid formation or UPS features
Chronic Osteomyelitis	Long latency (20–30 yr), foul odor, long-standing sinus tract	Irregular osteolysis, mixed lytic–sclerotic changes, enhancing mass at sinus tract	Squamous cell carcinoma from sinus epithelium
Post-Radiation Bone	Appears 5–20 yr post-radiation (>3000 cGy); new mass	New lytic lesion in irradiated field, cortical destruction, soft-tissue extension	High-grade sarcoma (OS/UPS) with radiation-related atypia
Bone Infarction	Long latency (10–20 yr), new or disproportionate pain	Loss of sclerotic rim, focal lysis, cortical destruction	Atypical spindle cells, osteoid/fibrous matrix
Paget’s Disease	New focal pain or mass in long-standing disease	Aggressive lytic lesion, cortical destruction, soft-tissue mass	Malignant osteoid → Secondary osteosarcoma; may also develop Paget-related GCT or hematologic malignancy
Low-Grade Central Osteosarcoma	Recurrence after incomplete resection/curettage within 3–5 yr	Mixed sclerotic–lytic intramedullary lesion → later permeative destruction and soft-tissue extension	Bland spindle cells with woven osteoid → dedifferentiation to high-grade OS (loss of MDM2/CDK4)
Low-Grade Conventional Chondrosarcoma	Dedifferentiation after long latency (>5 yr); sudden pain, or pathologic fracture	Lobulated lesion with ring-and-arc calcification → loss of matrix, cortical breakthrough, solid enhancement	Biphasic lesion: low-grade cartilage adjacent to high-grade UPS/OS component

**Table 2:** Clinical and Treatment-Related Risk Factors for Malignant Transformation.

Category	Key Examples	Mechanism/Risk Basis	Recommended Surveillance
Genetic syndromes	Multiple hereditary exostoses, Ollier disease, Maffucci syndrome	EXT or IDH mutations → abnormal cartilage growth with increased risk of secondary chondrosarcoma	Annual imaging (MRI or radiograph) and lifelong follow-up
Medication-related	Denosumab-treated GCTB	RANKL inhibition may induce aberrant osteoblastic proliferation and sarcomatous transformation (OS or UPS)	MRI every 6–12 months after cessation
Surgery-related	Marginal or intralesional excision of low-grade osteosarcoma or chondrosarcoma	Residual tumor cells may dedifferentiate into high-grade sarcoma	Long-term surveillance; recurrence within 3–5 years
Radiation-related	Bone within prior irradiation field (>3000 cGy)	Radiation-induced DNA damage and osteonecrosis	Begin MRI/CT at 5 years post-radiation; continue ≥ 20 years

## Data Availability

The data presented in this study are available on request from the corresponding author due to institutional and IRB-related restrictions.

## Abbreviations

CT	computed tomography
GCTB	giant cell tumor of bone
LGCOS	low-grade central osteosarcoma
LSMFT	liposclerosing myxofibrous tumor
MRI	magnetic resonance imaging
RIS	radiation-induced sarcoma
SCC	squamous cell carcinoma
SI	signal intensity
T1WI	T1-weighted image
T2WI	T2-weighted image
UPS	undifferentiated pleomorphic sarcoma
